# The Role of Gender in Nurse-Resident Interactions: A Mixed-methods Study

**DOI:** 10.5811/westjem.2021.3.49770

**Published:** 2021-07-19

**Authors:** Emily C. Cleveland Manchanda, Anita N. Chary, Noor Zanial, Lauren Nadeau, Jennifer Verstreken, Eric Shappell, Wendy Macias-Konstantopoulos, Valerie Dobiesz

**Affiliations:** * Massachusetts General and Brigham and Women’s Hospitals, Boston Harvard Affiliated Emergency Medicine Residency, Boston, Massachusetts; † Harvard Medical School, Program in Global Surgery & Social Change, Boston, Massachusetts; ‡ Massachusetts General Hospital, Department of Emergency Medicine, Boston, Massachusetts; § Brigham and Women’s Hospital, Department of Emergency Medicine, Boston, Massachusetts; ¶ Harvard Medical School, Department of Emergency Medicine, Boston, Massachusetts

## Abstract

**Introduction:**

The role of gender in interprofessional interactions is poorly understood. This mixed-methods study explored perceptions of gender bias in interactions between emergency medicine (EM) residents and nurses.

**Methods:**

We analyzed qualitative interviews and focus groups with residents and nurses from two hospitals for dominant themes. An electronic survey, developed through an inductive-deductive approach informed by qualitative data, was administered to EM residents and nurses. Quantitative analyses included descriptive statistics and between-group comparisons.

**Results:**

Six nurses and 14 residents participated in interviews and focus groups. Key qualitative themes included gender differences in interprofessional communication, specific examples of, and responses to, gender bias. Female nurses perceived female residents as more approachable and collaborative than male residents, while female residents perceived nurses’ questions as doubting their clinical judgment. A total of 134 individuals (32%) completed the survey. Females more frequently perceived interprofessional gender bias (mean 30.9; 95% confidence interval {CI}, 25.6, 36.2; vs 17.6 [95% CI, 10.3, 24.9). Residents reported witnessing interprofessional gender bias more frequently than nurses (58.7 (95% CI, 48.6, 68.7 vs 23.9 (95% CI, 19.4, 28.4). Residents reported that gender bias affected job satisfaction (P = 0.002), patient care (P = 0.001), wellness (P = 0.003), burnout (P = 0.002), and self-doubt (P = 0.017) more frequently than nurses.

**Conclusion:**

Perceived interprofessional gender bias negatively impacts personal wellbeing and workplace satisfaction, particularly among female residents. Key institutional stakeholders including residency, nursing, and hospital leadership should invest the resources necessary to develop and integrate evidence-based strategies to improve interprofessional relationships that will ultimately enhance residency training, work climate, and patient care.

## BACKGROUND

The importance of teamwork and interprofessional collaborative practice in clinical care cannot be overstated. Evidence suggests that gender has effects on the culture, practice, and organization of medicine for both nurses and physicians at all levels of training; these effects intersect with perceptions of power dynamics, professional hierarchies, and spheres of practice.^1^–^4^ The quality of nurse-physician interactions affects provider wellness in the workplace.^5^ Studies demonstrate that physicians’ and nurses’ perspectives differ with regard to both the quality of their interactions and the degree of interprofessional collaboration and respect.^4^–^8^ Further-more, evidence suggests that among medical students and resident physicians the perceived importance of collaborative interprofessional care may decrease over time.^9^ Interactions between female resident physicians and female nurses may be particularly challenging, as the intersection of gender and professional identities can lead to dysfunctional communication patterns.^10^

Effective communication and collaborative decision-making between nurses and physicians contributes to high-quality care, while poor team dynamics, disrespect, and miscommunication adversely affect patient safety^8^,^11^–^16^ and length of stay.^17^ Patient care “mishaps” may result from communication failures between nurses and residents.^18^ Developing strong interprofessional relationships may be particularly challenging for students^2^ and resident physicians,^10^ as they are by nature of their roles only transient members of clinical teams. While a clinician’s years of experience play a role in establishing positive interprofessional relationships,^12^ less is known regarding the role of gender, particularly for interprofessional relationships.

Gender disparities persist within the medical field for both nurses and physicians, with studies documenting continued salary disparities for both professions.^19^–^21^ There is also evidence of significant differences in faculty evaluation of female and male trainees with respect to milestone achievements during residency,^22^,^23^ which may be attributable to unconscious gender bias. Similarly, female gender is associated with more negative nursing evaluations of resident physicians;^24^,^25^ however, limited data exist to explain factors that contribute to this disparity.^26^ Research on the intersection of gender on resident/nursing interactions and leadership styles during resuscitations reveals that female residents express higher stress levels and discomfort when exhibiting directive leadership styles, despite this often being perceived as the most effective style; furthermore, female residents report needing to negotiate interactions, “gain trust,” or choose less assertive behaviors during interprofessional interactions than their male counterparts.^10^,^27^–^29^

However, the impact of gender bias on interprofessional relationships is not as well studied,^10^,^30^,^31^ in particular the extent to which gender bias occurs in interactions between resident physicians and nurses. During residency, physicians develop behavioral practice patterns that may last throughout their careers. The aim of this study was to explore and understand perceptions and experiences of gender bias in the context of interprofessional relationships between emergency medicine (EM) residents and nurses. This study builds on emerging literature exploring the ways in which gender shapes interactions between nurses and physicians during residency training.^10^ Our findings can inform strategies for improved interprofessional collaborative practice during residency training.

**Population Health Research Capsule**

What do we already know about this issue?

*Gender disparities persist in emergency medicine (EM). Gender shapes the culture, practice, and organization of medicine for both nurses and physicians*.

What was the research question?

*How does gender affect interprofessional interactions between EM resident physicians and nurses?*

What was the major finding of the study?

*Perceived interprofessional gender bias negatively impacts personal wellbeing and workplace satisfaction, particularly among female residents*.

How does this improve population health?

*Understanding how gender and gender bias affect interprofessional dynamics in EM creates opportunities to improve teamwork and patient care*.

### Study Objective

Our goal was to explore the effects of gender on interprofessional interactions between EM resident physicians and nurses.

## METHODS

This sequential mixed-methods study gathered qualitative data, which informed the development of a quantitative survey. The study was conducted at two urban, academic, Level I trauma centers with annual ED censuses of approximately 63,000 and 115,000. Spanning these two EDs is a single, four-year EM residency program that matches 15 residents per year.

### Phase I: Qualitative Study

We recruited EM nurses and resident physicians to participate in qualitative interviews and focus groups. We limited recruitment of resident physicians to second-, third-, and fourth-year residents given their longitudinal experiences with nursing colleagues. Similarly, nurses recruited for participation in the qualitative portion of this study were limited to those with more than two years of institutional experience. The research team’s resident members [EC, AC] contacted eligible participants from a roster of 42 residents, while the research team’s nurse members [JV, LN] contacted a convenience sample of 31 nurses from both institutions who were eligible and who had indicated in informal conversations that they would be willing to participate. After an individual expressed their willingness to participate, scheduling was taken over by the team’s social scientist [NZ], who conducted interviews and focus groups.

Semi-structured interviews were piloted with three individuals (one female nurse and two residents, one male and one female) to refine interview and focus group guides. Subsequently, focus groups were conducted with residents, separated by gender. Due to scheduling challenges, five nurses from two different institutions opted to participate in individual interviews rather than as part of a focus group. Between June–October 2019 interviews and focus groups were conducted by a trained interviewer [NZ] with no professional role in the residency or either ED. Questions focused on providers’ perceptions and experiences of how gender affects interprofessional interactions (Appendix 1). Interviews ranged from 20–40 minutes; focus groups lasted 90 minutes. Interviews and focus groups were recorded with consent of participants and transcribed verbatim using a transcription service (TranscribeMe, Inc., Oakland, CA).

We analyzed using inductive and thematic content analysis,^32^ allowing dominant themes to emerge. Free-text responses from the electronic survey (see below) were also coded and included in qualitative analysis. The research team developed a codebook from successive rounds of reviewing transcripts. Each transcript was coded for themes independently by two of four authors [EC, LN, JV, NZ] using a web-based, qualitative data analysis tool (Saturate, Jonathan Sillito, Brigham Young University, Provo, UT). An experienced qualitative researcher [AC] led resolution of coding discrepancies with research team input.

#### Reflexivity

Reflexivity in qualitative research refers to researchers’ consideration of how their sociocultural values and experiences influence study design and analysis. Qualitative data was collected by a social scientist [NZ] who does not have clinical EM experience. Analysts were all female, and included senior EM nurses [LN, JV], emergency physicians [AC, EC; both senior residents], and social scientists [AC, NZ]. Coding pairs were intentionally grouped across professions (nurse/MD, MD/social scientist or nurse/social scientist). Results were additionally presented to healthcare providers at three local and national conferences for feedback on interpretation of the major themes identified. To protect participant identity, transcripts are not publicly available. The study codebook is available in Appendix 2.

### Phase II: Quantitative Study

An anonymous electronic survey, developed through an inductive-deductive approach informed by the interviews and focus groups, was administered via Research Electronic Data Capture (REDCap, Vanderbilt University, Nashville, TN) and distributed via institutional email to all EM residents (60 individuals) and EM nurses at both hospitals (159 at one facility and 203 at the other), regardless of experience level. Up to two reminders of the invitation to participate were sent over the course of one week. Respondents were asked about the perceived frequency with which gender affects both their personal and witnessed interactions with colleagues across professions. Participants were also asked about how interprofessional gender bias affects the workplace with regard to job satisfaction, patient care, personal wellness, burnout, self-doubt, and patient safety. We collected basic demographic and professional experience data. Complete survey questions are available (Appendix 3).

For the purpose of exploring the impact of seniority on perceptions of gender bias, postgraduate year (PGY)-1 and -2 residents are considered “junior,” while PGY-3 and -4 residents are considered “senior.” Nurses with >4 years of experience were considered “senior,” while those with fewer years of experience were considered “junior.” We analyzed data in Stata 15.0 (StataCorp, LLC, College Station, TX), and included descriptive statistics and between-group comparisons using Student’s t-tests for continuous data and two-sample Wilcoxon rank-sum test for ordinal data.

#### Ethics

This study was reviewed by the local institutional review board and determined to be exempt from further review (Protocol #2019P000147). Funding to support this research was provided by the Massachusetts chapter of the American College of Emergency Physicians 2018 Resident Research Grant. We report qualitative findings following the Standards for Reporting Qualitative Research guidelines.^33^

## RESULTS

This study included 20 participants in the first, qualitative phase, and 134 respondents to the quantitative survey. The findings from each phase are described below, and in Tables 1–5.

### Qualitative Data

A total of 20 individuals participated in the qualitative portions of this study (see Table 1). Individual interviews were conducted with eight participants: six nurses (three female and three male, two from one institution and four from the other), and two residents (one female and one male). Focus groups were gender-specific with seven male residents in one group and five female residents in the other. Four themes emerged from qualitative data: communication in interprofessional relationships; specific examples of gender bias toward nurses; specific examples of gender bias toward residents; and responses to interprofessional gender bias. Representative quotes from each of these themes are listed in Table 2.

#### I. Communication

The theme of how gender shapes communication in interprofessional relationships emerged from data collected from both residents and nurses, particularly among females of each group. Examples of how gender shapes interprofessional communication significantly differed between nurses and residents. Female residents perceived questions from nurses about patient care as a threat to their decision-making and expertise. Female nurses identified feeling that female residents are more approachable about patient care questions and are more collaborative in their language and behavior than male residents.

#### II. Examples of Gender Bias toward Nurses

Nurses offered two major examples of witnessed or experienced interprofessional gender bias. They described male residents dismissing female nurses’ perspectives about patient care and emphasized that this occurs much more frequently than with female residents. Dismissive behaviors included residents not being willing to engage in conversation about nurses’ concerns about orders, lab values, or plans of care. The second example centered on the perception that male nurses receive more respect than female nurses. Both female and male nurses perceived that resident physicians, irrespective of the physician’s gender, take male nurses’ input about patient care more seriously.

#### III. Examples of Gender Bias toward Residents

When residents were asked for specific examples of witnessed or experienced gender bias toward residents, two major examples were described. First, female and male residents alike perceived that female residents receive more “pushback” from nurses of both genders. This included nurses questioning residents about orders and plans, disregarding residents’ plans, or not supporting residents in performing procedures. While residents held these perceptions regarding patient care in general, some reported the dynamics as more obvious and upsetting to female residents when they occurred during trauma and critical care resuscitations. Secondly, both female and male residents perceived that male residents have greater ease establishing friendly and collegial relationships with female nurses. With the exception of a few female nurses with whom male residents had difficult interactions, male and female residents perceived female nurses to be more friendly with male residents and interested in socializing with them outside the hospital. Female residents felt that they had to work harder and be more deferential toward female nurses to build relationships with them over time.

Participants from both professions recognized that gender alone did not account for their or others’ experiences of being dismissed or questioned. Rather, residents reported that gender had a lesser impact on interprofessional interactions as they progressed through training and gained more institutional experience.

#### IV. Responses to Gender Bias

Several suggestions emerged within the theme of responses to interprofessional gender bias. Both residents and nurses identified filing “safety reports,” the institutional standard for addressing quality concerns, as a potential course of action. However, residents identified their lack of anonymity as a major deterrent to pursuing this option. Nurses identified filing a complaint with the human resources department as an alternative. However, no respondents reported having taken these steps. Both nurses and residents gave examples of discussing biased interactions with their same-profession colleagues, including the emotional impact of these problematic experiences.

### Quantitative Survey

In total, 134 individuals (32% response rate) completed the survey, including 104 nurses (28.7% response rate) and 30 residents (52.6% response rate) (Table 1). Participating nurses were 84.6% female, while 36.7% of resident respondents were female. The gender balance of respondents roughly reflected that of each of these groups (approximately 80% female nurses at each institution; 38.5% of residents identified as female, yielding a 47.8% response rate among female EM residents). None of the respondents identified as non-binary. Among nurses, four individuals preferred not to indicate their gender, and their data were omitted from between-gender comparisons. The mean age of nurse respondents was significantly older than residents (36.7 vs 29.4 years, *P* <0.001). Most (80.6%) respondents self-identified as White, although the resident cohort had greater racial diversity (Table 1).

#### Perceptions of Gender Bias in Interprofessional Interactions

Perceptions of the frequency with which respondents both *experienced* and *witnessed* interprofessional gender bias were evaluated on a 100-point scale, labeled from “never” (0) to “always” (100) (Table 3). Among all respondents, females more frequently reported *experiencing* interprofessional gender bias than males (mean frequency 30.9, 95% confidence interval [CI], 25.6, 36.2 vs 17.6, 95% CI, 10.3, 24.9). This difference was noted both among female nurses (mean frequency 26.4, 95% CI 21.3, 31.4) vs 9.9 (95% CI, 2.5, 17.3] among male nurses, as well as among female residents (mean frequency 66.9 (95% CI 53.8, 80.0]) vs 22.5 (95% CI, 11.6, 33.4) for male residents. Female residents more frequently experienced interprofessional gender bias than their female nursing colleagues (mean frequency 66.9 [95% CI 53.8, 80.0] for female residents vs 26.4 [95% CI, 21.3, 31.4] for female nurses), but no significant difference was noted between male nurses and male residents.

Overall, significant between-profession differences emerged in the reported frequency of *witnessing* interprofessional gender bias. Resident physicians reported witnessing this bias more frequently than nurses [mean 58.7 (95% CI, 48.6, 68.7) among residents vs 23.9 (95% CI, 19.4, 28.4) among nurses [see Table 3]). This held true across both genders, such that female residents reported witnessing interprofessional gender bias more frequently than female nurses (mean 73.5 [95% CI, 57.3, 89.8] vs 23.4 [95% CI, 18.6, 28.3]), and male residents more frequently reported this than male nurses (50.1 [95% CI, 38.0, 62.1] vs 17.8 [95% CI, 3.6, 31.9]).

#### Perceived Manifestations of Interprofessional Gender Bias

Several questions explored the perceived manifestations of interprofessional gender bias that emerged from qualitative data, including the following: having a concern raised about oneself to a superior; having an order ignored; being given less trust; having one’s role confused by a cross-professional colleague; and being called a term of endearment by a cross-professional colleague. With the exception of having orders ignored, resident physicians reported experiencing each of these manifestations of gender bias significantly more frequently than their nursing colleagues (Table 4). No significant differences were identified between female and male nurses; however, female residents experienced each of these significantly more frequently than their male resident colleagues.

#### Impact of Gender Bias in Interprofessional Interactions

Respondents were asked about the frequency with which interprofessional gender bias affected several aspects of their work experience and patient care. Residents, when compared with nurses, more frequently felt gender bias negatively affected job satisfaction (*P* = 0.002), patient care (*P* = 0.001), personal wellness (*P* = 0.003), burnout (*P* = 0.002), and self-doubt (*P* = 0.017). Female residents felt gender bias affected these areas more frequently than their male colleagues, and more frequently than female nurses (Table 5). No significant between-gender differences were found among nurses on these factors, nor between male nurses and male residents.

Seniority did not modify any of the aforementioned relationships. The perceived negative impact of gender bias on job satisfaction increased with seniority among female residents (*P* = 0.01), but seniority was not otherwise associated with significant differences in the perceived impact of interprofessional gender bias.

## DISCUSSION

Gender shapes the professional experiences of healthcare providers, including medical students,^2^ resident physicians,^10^,^28^ and nurses.^20^,^21^ The extent to which gender bias shapes interprofessional interactions between residents and nurses remains incompletely described, although existing literature suggests that female gender identity may complicate interprofessional interactions.^10^ Power and privilege are created and justified through multiple social identities: Gender operates not alone but in conjunction with sexuality, race, ability, and other social identities to advantage some and disempower others.^34^ By design, this study focused specifically on the ways in which gender affects interprofessional interactions between resident physicians and nurses in the emergency department (ED).

Our study is situated in an understanding of gender through gender socialization theory,^35^ which posits that humans learn femininity and masculinity through social interactions, primarily with their families, peers, and groups. We become socialized into traditionally binary gender roles and identities, which create differential societal expectations for males’ and females’ behaviors. These expectations of gender roles permeate all environments, from the household to the workplace. In medicine, for example, women are expected to display caregiving and communicative capacities, while men are expected to display leadership and decision-making capacities, stemming from traditional gender roles within the household and society at large. Particularly early in residency, informal learning occurs in interprofessional relationships.^3^ This learning may shape long-standing behaviors and can affect professional identity development. As women now make up almost half of resident physicians across specialties,^36^ it is more important than ever to understand the ways that gender and gender bias affect interprofessional relationships.

This study reveals that both nurses and residents view gender as an important factor influencing interprofessional interactions; however, the perceived manifestations and impact of gender differed sharply between the two professional groups. This was most notable in qualitative data revealing how gender shapes communications between EM nurses and residents. While EM nurses expressed frustration with male residents, who were viewed as more dismissive and less collaborative when approached with a patient care question, female residents felt that frequent questioning of their clinical plans by nursing colleagues, and particularly from female nurses, reflected a lack of trust of female physicians. These starkly different perceptions of the same interactions build on prior literature demonstrating that physicians and nurses have disparate experiences of their interprofessional interactions with regards to communication and collaboration.^4^–^8^,^10^,^12^ While the intent behind nursing-initiated communication with residents is to improve patient care, this study revealed that for female residents in particular such interactions may increase self-doubt and insecurity. Understanding these differing perspectives highlights the need for further collaborative and longitudinal discussions between the two groups, particularly among females early in residency training, in order to bridge this gap and find ways to both mitigate problematic interactions and clarify the intent and goals of such conversations.^26^

Examples of gender bias shared by both residents and nurses reveal persistent and stark differences in how male and female health professionals experience the workplace. While males were more willing to attribute negative interprofessional interactions to personality differences, females more often identified gender as a defining factor in shaping these relationships. These findings were further underscored in the survey findings, as females of both professions reported experiencing interprofessional gender bias more frequently than their male counterparts (among female vs male nurses, mean frequency 26.4 [95% CI, 21.3, 31.4] vs 9.9 [95% CI, 2.5, 17.3] and among female vs male residents, 66.9 [95% CI, 53.8, 80.0] vs 22.5 [95% CI, 11.6, 33.4)]). Female residents reported both experiencing and witnessing interprofessional gender bias to a much greater degree. Female residents more frequently reported perceiving the various manifestations of gender bias in their cross-professional interactions (Table 4), and similarly were far more likely to report that this adversely affected their experiences in the workplace (Table 5).

The perceived negative impact of interprofessional gender bias on female residents in the ED may in part result from female residents taking on stereotypically gender-discordant professional roles,^26^,^37^–^39^ through which they are expected or encouraged to take on more typically male characteristics. The persistent and pervasive negative effects of gender bias in interprofessional interactions may have implications for patient care and patient safety. Effective communication across professional lines is a key component in the delivery of high-quality care; there is ample evidence that disrespect and poor team dynamics can harm patients.^8^,^11^–^16^

Across both professions, participants in the qualitative study expressed a sense of limited agency in addressing instances of perceived gender bias, which translated into a sense of apathy, frustration, or both. Both residents and nurses felt that additional years of experience may mitigate challenges in interprofessional interactions. Although the study was not primarily designed to explore the interaction between seniority and the various manifestations of interprofessional gender bias explored in this study, in quantitative analysis no such correlation emerged. Further investigation into the relationship between years of professional experience and the ways in which gender shapes interprofessional interactions may prove fruitful.

Educators and leaders within medicine may find it useful to look to the business world for examples of how gender shapes workplace interactions. Much has been written about gender and leadership in business, including the ways in which stereotypically female leadership styles, which in some sectors may be more democratic and participatory, rely more on communication and relationship building.^40^,^41^ Men may benefit from adopting some of these collaborative styles in business,^42^ and this may be true for clinicians of both genders in medicine. Relational coordination, a “mutually reinforcing process of communicating and relating for the purposes of task integration” described by Gittell et al, has proven effective in healthcare settings both for improving quality of care and job satisfaction among clinicians.^43^ Fostering strong interprofessional relationships between early-career physicians and nurses, particularly between females, may increase job satisfaction and mitigate perceptions of gender bias.

During residency, physicians not only learn medical knowledge and procedural skills, but also develop leadership styles and other patterns of behavior that can persist throughout their careers. Strong inter-professional relationships are integral to providing excellent patient care.^11^–^15^,^17^,^18^ Fostering collaborative interprofessional communication and strong nurse-physician relationships while in residency may result in attending physicians who promote and model more collaborative behaviors throughout their careers. Support from nursing leadership in EDs to foster positive, gender-informed interactions between EM nurses and the residents who work alongside them is equally important to fostering a collegial and respectful work environment for all healthcare providers. Educators and administrators – physicians and nurses alike – must consider and endeavor to understand the ways in which gender affects these interactions.^38^

Several strategies for improving interprofessional communication between residents and nurses have been explored by others, including structured “huddles,”^44^ simulation exercises,^45^ and collaborative “time-outs” prior to patient discharge,^46^ with variable efficacy. Further work is needed to understand, develop, and implement strategies for mitigating the negative impact of gender bias in interprofessional interactions; study participants suggested several possible interventions, which may warrant additional exploration through future research. Most of our study participants perceived gender bias in the clinical environment but demonstrated a reluctance to report this bias. Effective and safe mechanisms to report incidents and to ensure accountability and follow up of these occurrences should be explored.

In this study we identified a variety of suggestions for improving other aspects of interprofessional interactions. One interesting recommendation for improving cross-professional female allyship was to establish mentoring pairs between a female nurse and incoming female resident during intern orientation. Other means of increasing awareness could include workshops, video learning, and simulation exercises. At a local level, the findings from this study have led to the formation of a working group at one of our institutions, through which nurses and residents are exploring strategies for improving communication and assuring mutually respectful interactions. Further study of the effect of gender bias in interprofessional interactions between resident physicians and nursing colleagues should include the ways in which this occurs across specialties and throughout the career cycle of clinicians.

## LIMITATIONS

This study has several limitations. First, it was conducted only at large, urban, training hospitals hosting a single residency training program. Findings may not be transferrable to other training environments. The qualitative data was gathered by a social scientist unaffiliated with either the residency program or the hospitals; however, her gender (female) may have influenced the information shared by participants. Similarly, the qualitative analysis team included only female researchers, which inevitably shaped our interpretation of this data. Nursing perspectives were proportionally less represented in the qualitative portion of this study due to logistical challenges in recruitment. Similarly, response bias may have influenced our findings, particularly for the quantitative portion of this study. While the gender balance of respondents was similar to that of eligible participants in both professions, the opinions of study participants may differ significantly from those who chose not to respond to invitations for either interviews/focus groups or the emailed survey.

Lastly, our study included few participants whose backgrounds are historically and contemporarily under-represented in medicine. The intersection of race and sexuality with gender identity inevitably affected our findings; further study is warranted to understand the ways in which other forms of social identity influence interprofessional relationships.

## CONCLUSION

Gender shapes interprofessional interactions between resident physicians and nurses. The perception of gender bias contributes to dissatisfaction in the workplace, the effects of which are felt by both male and female nurses and residents, but disproportionately more by females of both professions. Female residents more frequently report experiencing the negative impacts of gender bias in their interprofessional relationships, raising concerns for their residency training and overall wellbeing. Key institutional stakeholders including residency, nursing, and hospital leadership should invest the resources necessary to develop and integrate evidence-based strategies to improve interprofessional relationships that will ultimately enhance residency training, work climate, and patient care.

## Supplementary Information



## Figures and Tables

**Table 1:** Demographics of participants by profession.

	Total	Nurses	Residents
Qualitative study			
Total participants	20	6	14
Interviews	8	6 (3F, 3M)	2 (1 M, 1 F)
Focus groups	12	0	12 (5F, 7M)
Survey respondents			
	
	N (%)	N (%)	N (%)
	
Complete responses	134 (32.0)	104 (28.7)	30 (52.6)
Gender			
Female	99 (73.9)	88 (84.6)+	11 (36.7)++
Male	31 (23.1)	12 (11.5)	19 (63.3)
Prefer not to say	4 (3)	4 (3.85)	0 (0)
	
	Mean (SD)	Mean (SD)	Mean (SD)
	
Age	36.7 (±10.15)	38.8 (±10.6)	29.4 (±2.2)
Tenure	N (%)	N (%)	N (%)
PGY1/<1 year	11 (8.2)	2 (1.9)	9 (30)
PGY2/1–2 years	23 (17.2)	13 (12.5)	10 (33.3)
PGY3/2–3 years	22 (16.4)	15 (14.4)	7 (23.3)
PGY4/3–4 years	10 (7.5)	6 (5.8)	4 (13.3)
>4 years	68 (50.8)	68 (65.4)	n/a
Race			
White	108 (80.6)	88 (84.6)	20 (66.7)
Black	7 (5.2)	6 (5.8)	1 (3.3)
Asian	6 (4.5)	0	6 (20.0)
American Indian	2 (1.5)	0	2 (6.7)
Other/prefer no reply	11 (8.2)	10 (9.6)	1 (3.3)
Hispanic	4 (3)	1 (1.0)	3 (10)

+ Approximately 80% of emergency medicine (EM) nurses in the study population identify as female; exact numbers were not available as this was beyond the scope of IRB-approved data collection.

++ At the time of this study, 23 of 60 EM residents identified as female (gathered through personal correspondence with ECM and AC), yielding a 47.8% response rate among female EM residents.

*M*, male; *F*, female; % respondents indicates response rate within professional group; *SD*, standard deviation; *PGY*, post-graduate year.

**Table 2:** Representative quotes.

Theme	Quote
I. Gendered communication in interprofessional relationships	
Differential communication strategies enacted by female residents	“I think the one thing that I have become particularly cognizant of is that the female residents almost always, when they place orders, will then go and talk to the nurse and tell them what orders they’ve placed, which I assume is a strategy they’ve developed to just actually enact the plans they want to happen.” - Male resident [Interview 1] And I went into the room, I saw that the patient was unstable, and I said-- or could be unstable. And I said, “Hey, do you think we should--” and it’s never a, “Do this,” it’s always like, “We should--” or “Can we--” The way we phrase things is also very different. I imagine it probably varies between men and women. But I think, again, going back to that-- you have to kind of do a shared decision making. I’m never commanding anybody to do anything, so it’s like, “Do you think we should get more IV access on this patient?” and she was just like, “No. This patient is totally stable, doesn’t need it. I’m not doing it.” - Female resident [Focus Group 1, speaker 4]
Female and male nurse interactions with female residents vs male residents	“Well, when I’ll question a dosing of a medication and asking for an explanation and if feels like-- I don’t want to put words in someone else’s mouth but I think sometimes the male doctor has maybe kind of sometimes brushed me off and sometimes explained but maybe not in a thorough way that I would like. Whereas I feel like some of the female residents have been more open to explaining the situation and their rationale and they go into more in depth and stuff than maybe some of the male doctors have and stuff. Where they have been more dismissive at times and stuff about why they’re doing things and stuff.” - Male nurse [Interview 4] “And for the most part, there’s always going to be somebody -- there’s always new and up-and-coming residents that will turn to the nurse and say, “What do you think?” And there have been. And in that case, I will say I’ve had more female residents ask me that than the male docs. I mean, there’s one here and there. Don’t get me wrong. But more of the female residents will say, “What do you think?” - Female nurse [Interview 5]
II. Examples of gender bias toward nurses	
Dismissal of female nurse’s concerns about patient care	“I’ve been called sweetie, hon, etc., more times than I can count. Been referred to as ‘just a nurse,’ and my input regarding patient care, decision-making or patient’s condition has been dismissed.” -Female nurse [Survey, open response, respondent 70] “I think sometimes the male doctor has maybe kind of brushed me off, and sometimes explained but maybe not in a thorough way that I would like. Whereas I feel like some of the female residents have been more open to explaining the situation and their rationale, and they go into more in depth and stuff than maybe some of the male doctors have.” -Male nurse [Interview 4] “So let’s say they put in an order and you disagree with it…Guy doctor will get all offended, not change it. You have to go above him usually and go to an attending in order to advocate for your patient, while a female resident will be like, ‘You know what? Thank you. I’m new to this. Let me look into what it was. I’ll double-check with my attending to make sure,’ and all that, instead of immediately being, ‘No. I’m right.’” -Male nurse [Interview 6] “As a female RN, I sometimes feel like some male physicians will not make eye contact with me when I am asking for a med request or patient questions, rather than females, who usually do look me in the eye. I do understand that we are all busy and focused on documenting and charting, but I feel disrespected when that eye contact is inconsistent.” -Female nurse [Survey, open response, respondent 145]
Preferential treatment for male nurses	“I would say that [male nurses] get taken more seriously [than female nurses] and that they’re not questioned as much about things that they say or feel.” -Female nurse [Interview 3] “As a male nurse, I feel that I am more frequently listened to by male physicians.” -Male nurse [Survey, open response, respondent 106]
III. Examples of gender bias toward residents	
Disproportionate pushback against female residents	“I think their orders are frequently questioned and their care plans are frequently questioned in a way that male residents are not.” -Male resident [Interview 1] “I think the interactions between the female and male residents and the nurses is different in the sense that you [as a female resident] get, I think, more pushback from nurses with orders, more questioning your judgment, more hesitancy in carrying out orders and doing tasks.” -Female resident [Focus Group 1, speaker 2] “I think I’ve probably seen more of the female nurses being more aggressive towards some of the female residents than male residents, I think in general. I think I’ve seen them be more critical of their same gender, so.” Male nurse [Interview 4] “I think that I commonly get more preferential treatment from nurses because I’m male. I think that especially within—a great example is when nurses will say that they have more confidence in your decision-making than they do in one of your female colleague’s decision-making.” -Male resident [Focus Group 2, speaker 2]
Building relationships with female nurses	“I can think of one specific example where the [male resident] got a little love note from a nurse basically saying, ‘Oh thank you for saving this patient’s life,’ with a little heart on the bottom…I just can’t imagine that happening with one of the women residents.” -Male resident, [Focus Group 2, speaker 4] “I certainly feel like male colleagues get a differential relationship and experience with the nurses—on attention to orders, attention to personal relationships, in a lot of ways, of trying to be much friendlier or more than friendly with male residents in a way that they’re less open to with female residents. And I don’t think that that’s always true across the board, but I see it more than I do with female residents.” -Female resident [Focus Group 1, speaker 5] “…[Male residents] can be friends [with nurses], but in moments of leadership, they can still be looked at as leaders. Whereas, I think a lot of times, the nurses don’t necessarily see the women as leaders; they’ll see them as peers.” -Female resident [Focus Group 1, speaker 4]
IV. Responses to gender bias	
Speaking with administration on issues of gender bias	“I’ve also brought this issue up to one of our administrative people, who’s higher up, here at the Brigham, and he was aware of the issue. And one of the feedback that I got was that I should be delegating more tasks to the nurses because he saw me bringing a CD to radiology and instead of doing that, I should be giving it to the nurse so that I can be at the bedside taking care of the patient. And when I try to explain, “As a female, it’s really hard to do,” and I don’t know if our male colleagues feel the same way, but--actually, it’s funny.” - Female resident [Focus Group 1, speaker 3]
Perceptions of the effectiveness of safety reports	“I don’t know, although I don’t know what happens to any of the safety reports that we do on anything. I think that, from my perspective as a resident, they seem fairly ineffectual. And I’m sure that’s not actually true. I’m sure there is some work that gets done on them. But I feel like for all the safety reports that people have been involved in, I’ve never actually noticed anything change in any way.” - Male resident [Interview 1]
Female nurse experiences with having gender bias addressed	“I think that if I said that I felt like he wasn’t taking me seriously because I was a girl, it probably would have been pushed under the rug, and that it would be taken more seriously if it was more advocating on the behalf of a patient. S1: 13:42 Do you know why that’s the case? S2: 13:43 I just don’t think they take it serious. I don’t think that it’s management’s prerogative to take that seriously.” - Female nurse [Interview 3]
Male nurses experiences of speaking about gender bias with female colleagues	“I’ve chatted with a few of them [female nurse colleagues], just about how disappointed they are, and not only just the few numbers of females nurses of color on our department, but just how little sway they have in the department in general.” - Male nurse [Interview 6]

**Table 3:** Perceptions of gender bias in interprofessional interactions.

	All	Nurses	Residents

	Mean* (95% CI)	Mean (95% CI)	Mean (95% CI)
Frequency of experiencing interprofessional gender bias			
All	29.6 (25.4, 33.8)	24.8 (20.3, 29.4)	38.8 (27.4, 50.1)
Female	30.9 (25.6, 36.2)	26.4 (21.3, 31.4)	66.9 (53.8, 80.0)
Male	17.6 (10.3, 24.9)	9.9 (2.5, 17.3)	22.5 (11.6, 33.4)
Frequency of witnessing interprofessional gender bias			
All	31.7 (26.9, 36.5)	23.9 (19.4, 28.4)	58.7 (48.6, 68.7)
Female	29 (23.5, 34.5)	23.4 (18.6, 28.3)	73.5 (57.3, 89.8)
Male	37.5 (27.1, 48.0)	17.8 (3.6, 31.9)	50.1 (38.0, 62.1)

* Values are a numeric representation of frequency on a 100-point scale, with 0 reflecting never and 100 reflecting always.

*CI*, confidence interval.

**Table 4:** Perceived manifestations of gender bias in interprofessional interactions.

	Between-profession comparison						Between-gender comparison			

	Nurses vs residents		Female nurse vs female resident		Male nurse vs male resident		Female vs male nurses		Female vs male residents	

	Z*	P	Z*	P	Z*	P	Z*	P	Z*	P
Called term of endearment	−5.84	<0.01	−5.08	<0.01	−2.765	0.01	−0.144	0.88	2.054	0.04
Role confused	−3.15	<0.01	−3.74	<0.01	−0.888	0.38	−0.281	0.78	2.304	0.02
Given less trust	−2.988	<0.01	−5.172	<0.01	−0.544	0.59	0.442	0.66	4.279	<0.01
Order ignored	0.21	0.83	−2.219	0.03	0.968	0.33	0.494	0.62	2.902	<0.01
Concern raised to attending	−6.887	<0.01	−6.163	<0.01	−2.6	0.01	−0.948	0.34	2.517	0.01

* Two-sample Wilcoxon rank-sum tests for between-group comparisons

**Table 5:** Perceived impact of gender bias in interprofessional interactions.

	Between-profession comparison						Between-gender comparison			

	Nurses vs residents		Female nurse vs female resident		Male nurse vs male resident		Female vs male nurses		Female vs male residents	

	Z*	P	Z*	P	Z*	P	Z*	P	Z*	P
Job satisfaction	−3.04	0.002	−4.39	<0.001	−1.15	0.250	0.85	0.400	3.50	<0.001
Patient care	−3.26	0.001	−3.98	<0.001	−1.83	0.068	1.39	0.166	2.40	0.016
Wellness	−2.96	0.003	−4.24	<0.001	−1.25	0.210	1.21	0.225	3.31	0.001
Burnout	−3.07	0.002	−4.41	<0.001	−1.08	0.280	0.71	0.478	3.17	0.002
Self-doubt	−2.39	0.017	−3.93	<0.001	−1.60	0.111	1.84	0.065	3.21	0.001
Patient safety	0.78	0.437	−0.95	0.344	−0.35	0.730	0.08	0.940	0.52	0.601

* Two-sample Wilcoxon rank-sum tests for between-group comparisons, all P-values two-tailed.
