# Supplementary material for: The Role of Gender in Nurse-Resident Interactions: A Mixed-methods Study
Source: West J Emerg Med. 2021 Jul 19;22(4):919–30. doi: 10.5811/westjem.2021.3.49770 (PMC8328169; doi:10.5811/westjem.2021.3.49770)
Supplement: Supplementary file 1 [file wjem-22-919-s001.docx]

**Appendix 1: Sample questions from interviews and focus groups**

| Resident Interview Guides |
| --- |
| 1. Have you experienced gender bias during residency training? How so?   *Probe:  Can you think of examples where you experience discrimination OR preferential treatment based on your gender identity?* |
| 1. Have you witnessed colleagues experience gender bias during residency training? How so? |
| 1. How have your interactions with nursing colleagues changed during residency?   *Probes:*  *Have you noticed differences in the way nurses treat you from intern year until now?*  *How collegial do you feel your relationships are with nurses now? Has this changed over time?* |
| 1. Have you ever reported mistreatment on the basis of gender? How was that experience? Were you satisfied with the response? Have you ever had difficulty knowing who to approach about this? |
| 1. What changes would you like to see to work towards more equitable clinical practice across the gender spectrum? |
| Nurse Interview Guides |
| 1. Are there differences in how male and female residents communicate with you? Are there differences in how you communicate with male and female residents? |
| 1. As a nurse, have you experienced gender bias from residents? How so?   *Probe:  Can you think of examples where you experienced discrimination OR preferential treatment based on your gender identity?* |
| 1. How have your relationships with residents changed over time as they progress through residency?   *Probes:*  *Have you noticed differences in the way residents treat you from intern to senior year?*  *How collegial do you feel your relationships are with residents now? Has this changed over time?* |
| 1. Have you ever reported mistreatment on the basis of gender? How was that experience? Were you satisfied with the response? Have you ever had difficulty knowing who to approach about this? |
| 1. What changes would you like to see to work towards more equitable clinical practice across the gender spectrum? |

**Appendix 2: Codebook for qualitative data analysis**

| Code | | Definition |
| --- | --- | --- |
| Level of awareness | Description of the level of awareness participants have around gender bias, including perceptions of others’ levels of awareness. Can include statements on *not* being aware or thinking of gender bias. | |
| Communication | Perception of how gender affects communication between nurses and residents. | |
| Gender bias towards resident | Descriptions of gender bias towards resident physicians *only* from nurses, not including other healthcare providers or patients. | |
| Gender bias towards nurse | Descriptions of gender bias towards nurse *only* from resident physicians, not including other healthcare providers or patients. | |
| Differential treatment based on level of experience | Account of how differences in interprofessional interactions are driven by level of provider (resident physician or nurse) experience. | |
| Responding to gender bias | How participants respond to gender bias personally and supporting others who have experienced it. Includes descriptions of reporting mechanisms and comfort level with these mechanisms. | |
| Suggestions for change | Specific ways in which gender bias in the workplace can be addressed as suggested by participants. | |

**Appendix 3: Electronic survey questions**

1. What is your clinical role? (Nurse/Resident physician)
2. At which institution(s) do you currently work? (Choose one or both)
3. [How long have you worked at this institution/What is your PGY-year?]
   1. Less than 1 year / PGY-1
   2. 1-2 years / PGY-2
   3. 2-3 years / PGY-3
   4. 3-4 years / PGY-4
   5. More than 4 years
4. What is your age in years?
5. What is your gender? (Female, Male, Other, prefer not to say)
6. Are you Hispanic or of Latinx descent? (yes/no/prefer not to say)
7. Please indicate your race (choose more than one as needed)
   1. White
   2. Black/African American
   3. Asian
   4. Native Hawaiian or other Pacific Islander
   5. American Indian or Alaska Native
   6. Other
   7. Prefer not to answer
8. To what degree do you feel that you experience gender-based discrimination or bias directed at you from a [nurse/resident physician]? (sliding scale, 0-100)

0 = No experience
50 = Some experience
100 = Very frequent experience

1. If you have personally experienced gender-based discrimination or bias within your work setting that came from a [nurse/resident physician], how often have you experienced the following types of discrimination? Please select "No experience" if you have not experienced that type of discrimination or bias from a [nurse/resident physician]. [Likert scale 1-5]
   1. A negative or inappropriate exchange occurred with a [nurse/resident physician] of your same gender
   2. A [nurse/resident physician] referred to you with an unprofessional and unwelcome "term of endearment". This may include but is not limited to: hon, honey, sweet, sweetheart, sweetie, darling, dear, babe, stud, bud, or buddy
   3. A [nurse/resident physician] mistook you for a non-physician or non-nurse member of the team, such as a housekeeping employee, a food service employee, a student, or a physical therapist
   4. A [nurse/resident physician] gave you less trust based upon your gender
   5. A [nurse/resident physician] ignored your request/recommendation regarding a patient-related task or order
   6. A [nurse/resident physician] spoke to the attending physician responsible for a shared patient to express concern about your role in patient care (e.g., a particular order or clinical decision)
2. To what degree do you feel that you have personally WITNESSED (i.e. occurring to someone else) gender-based discrimination or bias coming from a [nurse/resident physician] in your workplace?

0 = No experience
5 = Some experience
10 = Very frequent experience

1. How do you introduce yourself to a patient? You may select more than one answer.
   1. Dr. [Last Name] 🡪 omit for nurses
   2. [First Name] [Last Name]
   3. Dr. [First Name] [Last Name] 🡪 omit for nurses
   4. [First Name]
   5. Other: please describe
2. Think back to the last time you introduced a male [nurse/resident physician] to a shared patient with whom you had already begun speaking when he [the (nurse/resident)] entered the room. How did you introduce him to the patient? For example, how did you finish the phrase, “This is…”
   1. Dr. [Last Name] 🡪 omit for resident survey
   2. [First Name] [Last Name]
   3. Dr. [First Name] [Last Name] 🡪 omit for resident survey
   4. [First Name]
   5. By title (e.g., “the resident” or “your nurse”)
   6. Other: please describe
3. Think back to the last time you introduced a female [nurse/resident physician] to a shared patient with whom you had already begun speaking when she [the (nurse/resident)] entered the room. How did you introduce her to the patient? For example, how did you finish the phrase, “This is…”
   1. Dr. [Last Name] 🡪 omit for resident survey
   2. [First Name] [Last Name]
   3. Dr. [First Name] [Last Name] 🡪 omit for resident survey
   4. [First Name]
   5. By title (e.g., “the resident” or “your nurse”)
   6. Other: please describe
4. Please indicate how you believe that the following factors have been affected by your personal experience(s) with gender-based discrimination or bias from [nurses/resident physicians] in your workplace. [5-point Likert, from not at all, to very much]
   1. Job satisfaction
   2. Quality of patient care
   3. Personal well-being
   4. Personal risk of burn-out
   5. Self-doubt
   6. Personal safety
5. This is the last question. Please use this space to share examples or stories of personally experienced or observed gender-based discrimination or bias in interactions between nurses and resident physicians in your workplace. What were the effects or consequences of the experience on you, on other members of the healthcare team, or on other patients? Please do not include personally identifiable information about yourself or others. [free text]
